# AP-2ε Expression in Developing Retina: Contributing to the Molecular Diversity of Amacrine Cells

**DOI:** 10.1038/s41598-018-21822-y

**Published:** 2018-02-21

**Authors:** Saket Jain, Darryl D. Glubrecht, Devon R. Germain, Markus Moser, Roseline Godbout

**Affiliations:** 1 Department of Oncology, University of Alberta, Cross Cancer Institute, Edmonton, Alberta Canada; 2 Max-Planck-Institute of Biochemistry, Martinsried, Germany

## Abstract

AP-2 transcription factors play important roles in the regulation of gene expression during development. Four of the five members of the AP-2 family (AP-2α, AP-2β, AP-2γ and AP-2δ) have previously been shown to be expressed in developing retina. Mouse knockouts have revealed roles for AP-2α, AP-2β and AP-2δ in retinal cell specification and function. Here, we show that the fifth member of the AP-2 family, AP-2ε, is also expressed in amacrine cells in developing mammalian and chicken retina. Our data indicate that there are considerably fewer AP-2ε-positive cells in the developing mouse retina compared to AP-2α, AP-2β and AP-2γ-positive cells, suggesting a specialized role for AP-2ε in a subset of amacrine cells. AP-2ε, which is restricted to the GABAergic amacrine lineage, is most commonly co-expressed with AP-2α and AP-2β, especially at early stages of retinal development. Co-expression of AP-2ε and AP-2γ increases with differentiation. Analysis of previously published Drop-seq data from single retinal cells supports co-expression of multiple AP-2s in the same cell. Since AP-2s bind to their target sequences as either homodimers or heterodimers, our work suggests spatially- and temporally-coordinated roles for combinations of AP-2 transcription factors in amacrine cells during retinal development.

## Introduction

The vertebrate retina consists of diverse neuronal cell types which coordinate the reception, processing and transfer of the visual signal to the optic centers in the brain. There are six major neuronal cell types (rod and cone photoreceptors, horizontal cells, bipolar cells, amacrine cells and ganglion cells) in the retina. Retinal progenitor cells give rise to these different neuronal cell types as well as Müller glial cells in a highly-coordinated manner governed by both extrinsic and intrinsic factors^1,2^. Retinal development can be broadly divided into three phases: cell proliferation, migration (exit from the cell cycle/lineage commitment), and differentiation. Transcription factors play important roles in all aspects of retinal development^3^.

AP-2 is a family of transcription factors involved in the regulation of genes responsible for cellular growth and differentiation during early development^4–6^. Overlapping and divergent AP-2 expression patterns suggest both redundant and non-redundant roles for AP-2 family members. In mice, AP-2α, AP-2β and AP-2γ are expressed in neural crest cell lineages, the peripheral nervous system, facial and limb mesenchyme, the epithelia of the developing embryo and/or extraembryonic trophectoderm^7–9^. AP-2δ is expressed in developing heart and CNS^10^ and AP-2ε expression was first reported in the olfactory bulb^11,12^ and keratinocytes^13^. *AP-2α* knockout mice die perinatally with craniofacial defects and severe skeletal defects in head and trunk regions^14,15^. *AP-2β* knockout mice die shortly after birth due to polycystic kidney disease and terminal renal failure^16,17^. *AP-2γ* knockout mice die during early embryonic development immediately after implantation^9,18^. Both *AP-2δ* and *AP-2ε* knockout mice are viable, with defects in midbrain development^19,20^ and olfactory bulb formation^21^, respectively.

AP-2 transcription factors play a significant role in eye development^22–25^, with four AP-2s (α, β, γ and δ) expressed in developing retina^25–29^. *AP-2α* and *AP-2β* are restricted to amacrine cells and horizontal cells^28,29^. AP-2γ is also expressed in amacrine cells, but in a population that is distinct from that of AP-2α and AP-2β^26^, whereas *AP2δ* is found in a subset of ganglion cells^30^. Conditional (retina-specific) *AP-2α* and *AP-2β* knockout mice show horizontal and amacrine cell defects that were not observed upon deletion of *AP-2α* alone^26,27^. This suggests redundant roles for AP-2α and AP-2β in amacrine and horizontal cell differentiation. In addition to midbrain defects, *AP2δ*-knockout mice also show reduced ganglion cell numbers as well as reduced axonal projections to the superior colliculus, a major visual center in the brain^20^. In chick retina, overexpression of AP-2δ results in axonal misrouting^31^.

Amacrine cells, distributed in the innermost part of the inner nuclear layer of the retina, are interneurons that form synapses with ganglion and/or bipolar cells, with key roles in the processing of visual signals^32,33^. Amacrine cells are the most diverse type of cells in the retina with >30 subtypes characterized to date^32,34^. Here, we report that AP-2ε is expressed in amacrine cells in chicken, mouse and human fetal retina. Immunofluorescence analysis reveals co-localization of AP-2ε with the other members of the AP-2 family, but only in subsets of cells and at specific developmental stages. Our results indicate highly specific and cell-restricted roles in the retina for this latest member of the AP-2 family. Since AP-2s can function as either homodimers or heterodimers, expression of four AP-2s in subsets of amacrine cells has implications for finely-tuned regulation of AP-2 target genes.

## Results

### *AP-2ε* is expressed in amacrine cells

**Figure 1:**
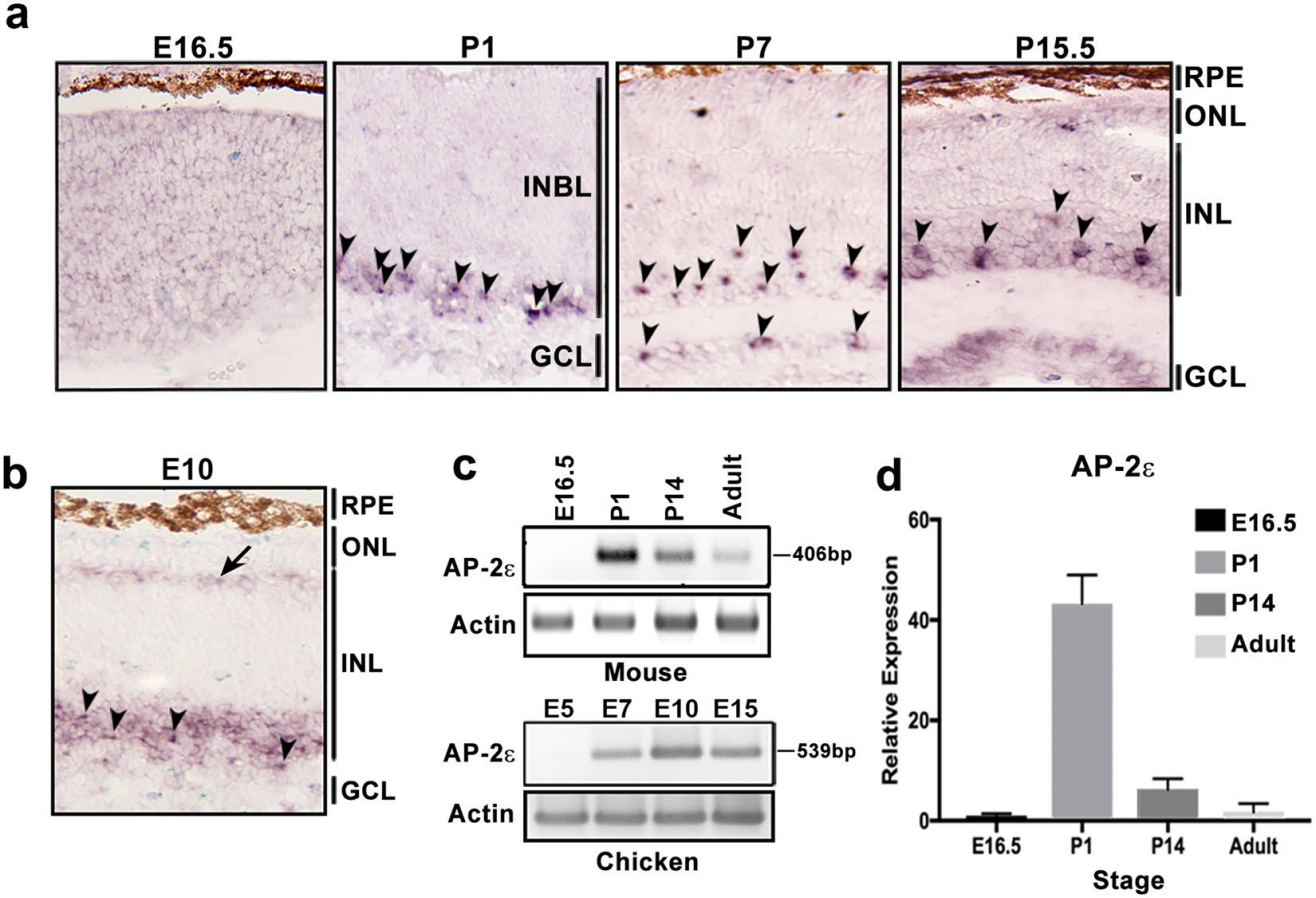
*AP-2ε* RNA is expressed in mouse and chick retina. (**a**) *In situ* hybridization showing expression of *AP-2ε* at E16.5, P1, P7 and P15.5 in mouse retina. (**b**) *In situ* hybridization showing expression of *AP-2ε* in E10 chick retina. (**c**) RT-PCR analysis of *AP-2ε* in mouse retina at E16.5, P1, P14 and adult (top), and in chick retina at E5, E7, E10 and E15 (bottom). Sizes of RT-PCR products are indicated on the right. Full length blots are shown in Supplementary Fig. S1. (**d**) qPCR analysis showing relative expression of *AP-2ε* in mouse retina at E16.5, P1, P14 and adult. The error bars are calculated using standard deviation. Arrowheads point to positive amacrine cells. The arrow points to the horizontal cell layer. Abbreviations: RPE, retinal pigmented epithelium; INL, inner nuclear layer; ONL, outer nuclear layer; GCL, ganglion cell layer; INBL, inner neuroblastic layer.

Expression of four members of AP-2 family has previously been documented in the developing retina, with AP-2α, AP-2β and AP-2γ all expressed in amacrine cells. We examined whether *AP-2ε* might also be expressed in the retina by carrying out *in situ* hybridization of mouse retinal tissue sections at E16.5 (mostly proliferative cells), P1 (early stage of differentiation), P7 (intermediate stage of differentiation) and P15.5 (late stage of differentiation). Only background staining was observed at E16.5, indicating that *AP-2ε* is not expressed in proliferating cells (Fig. 1a). By P1, *AP-2ε* RNA was detected in the inner part of the inner neuroblastic layer where amacrine cells are located. At P7 and P15.5, there were *AP-2ε*-positive cells throughout the inner part of the inner nuclear layer, along with a few positive cells in the ganglion cell layer of P7 retina. The *AP-2ε* distribution patterns at P1, P7 and P15.5 are consistent with expression in amacrine cells, as displaced amacrine cells are also found in the ganglion cell layer.

We then examined whether *AP-2ε* expression in amacrine cells is evolutionarily conserved. *In situ* hybridization of chick retina tissue sections was carried out at E10 which is roughly equivalent to mouse P7 retina^35,36^. Similar to mouse, *AP-2ε* RNA in chick retina was found in the amacrine cells located in the inner part of the inner nuclear layer (indicated by arrowheads in Fig. 1b). No signal was observed in the ganglion cell layer, likely reflecting the reduced numbers of displaced amacrine cells in the ganglion cell layer of chick retina compared to mouse retina^37,38^. However, there was a layer of *AP-2ε-*positive cells in the outer part of the inner nuclear layer where horizontal cells are located (indicated by arrow in Fig. 1b).

To confirm our findings that AP-2ε is expressed in both mouse and chicken retina, we carried out RT-PCR analysis at different stages of development. In mouse retina, no signal was detected at E16.5, in agreement with the *in situ* hybridization data (Fig. 1c and Supplementary Fig. S1). A strong signal was obtained in P1 retina, with progressively weaker signals in P14 and adult retina. These semi-quantitative data were verified by quantitative RT-PCR (Fig. 1d). In chick retina, no signal was detected in the relatively undifferentiated E5 retina, with a peak signal observed in E10 retina (Fig. 1c and Supplementary Fig. S1).

**Figure 2:**
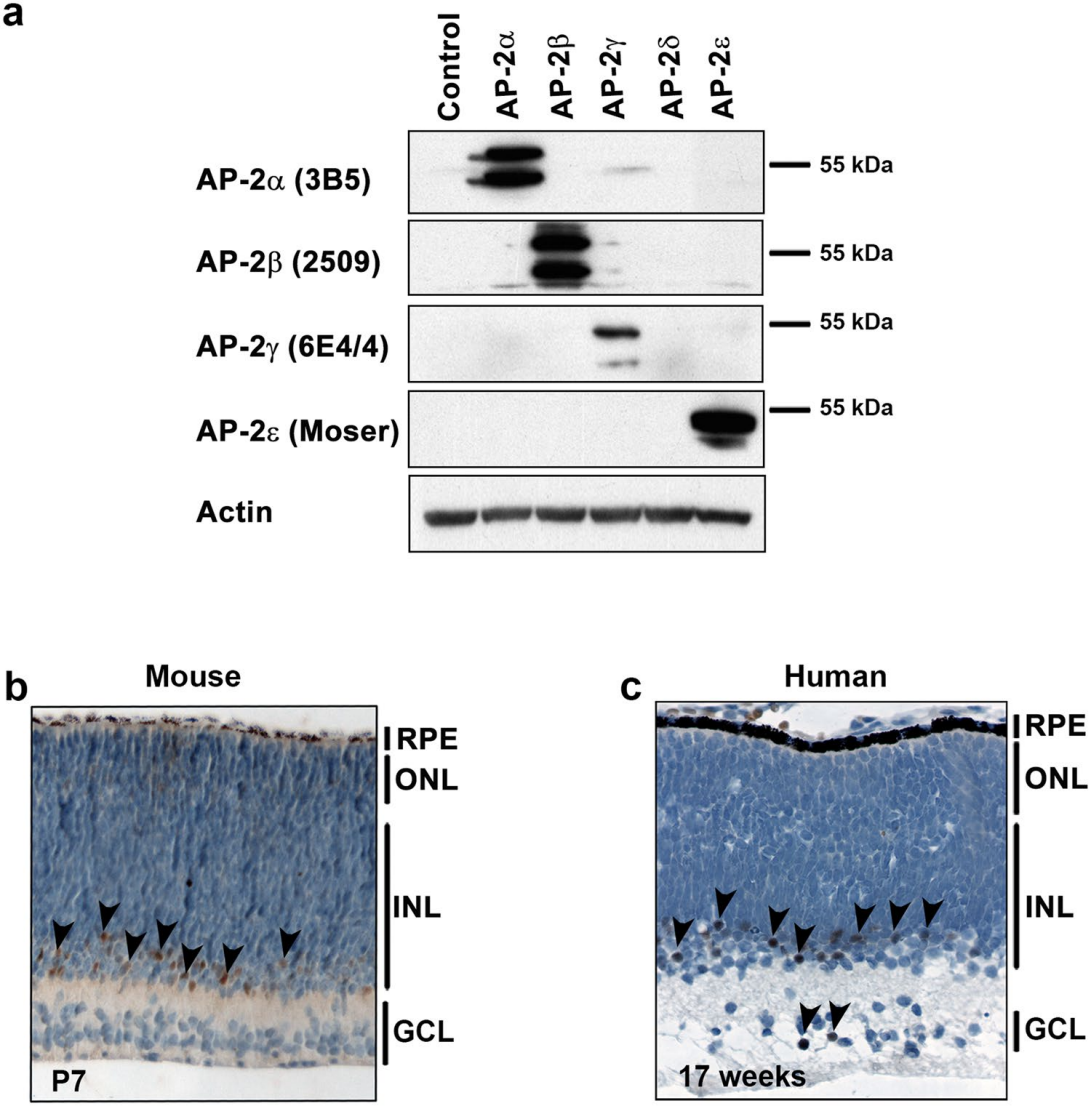
Immunohistochemical analysis of AP-2ε in retina. (**a**) Western blot analysis of AP-2 antibodies. HeLa cells were transfected with vector control, AP-2α, AP-2β, AP-2γ, AP-2δ or AP-2ε expression constructs. Blots were immunostained with antibodies to AP-2α, AP-2β, AP-2γ or AP-2ε. Full-length blots are presented in Supplementary Fig. S2. (**b**) P7 mouse retina and (**c**) human fetal retina at 17 weeks gestation were immunostained with the anti-AP-2ε antibody. Positive cells are indicated by arrowheads. Abbreviations: RPE, retinal pigmented epithelium; INL, inner nuclear layer; ONL, outer nuclear layer; GCL, ganglion cell layer.

Next, we carried out immunohistochemical analysis to examine the distribution of AP-2ε protein in retina. We first tested the specificity of our AP-2 antibodies by western blot analysis of HeLa cells transfected with different AP-2 expression constructs. Based on western blotting, the AP-2α, AP-2β, AP-2γ and AP-2ε antibodies are highly specific (Fig. 2a and Supplementary Fig. S2). The presence of doublet bands suggests post-translational modification of AP-2 proteins. We then used the AP-2ε antibody to immunostain mouse retina. In P7 mouse retina, AP-2ε-positive cells were observed in the inner nuclear layer (arrowheads point to positive cells) (Fig. 2b). We also examined the distribution of AP-2ε in human fetal retina at 17 weeks gestation, a stage when amacrine cells are differentiated^39^. Similar to what we observed in mouse retina, AP-2ε-positive cells in human retina were mostly confined to the inner part of the inner nuclear layer where amacrine cells are located (Fig. 2c). A few AP-2ε-positive cells were also found in the ganglion cell layer, likely displaced amacrine cells.

### Co-expression of AP-2ε and other AP-2 family members in retina

**Figure 3:**
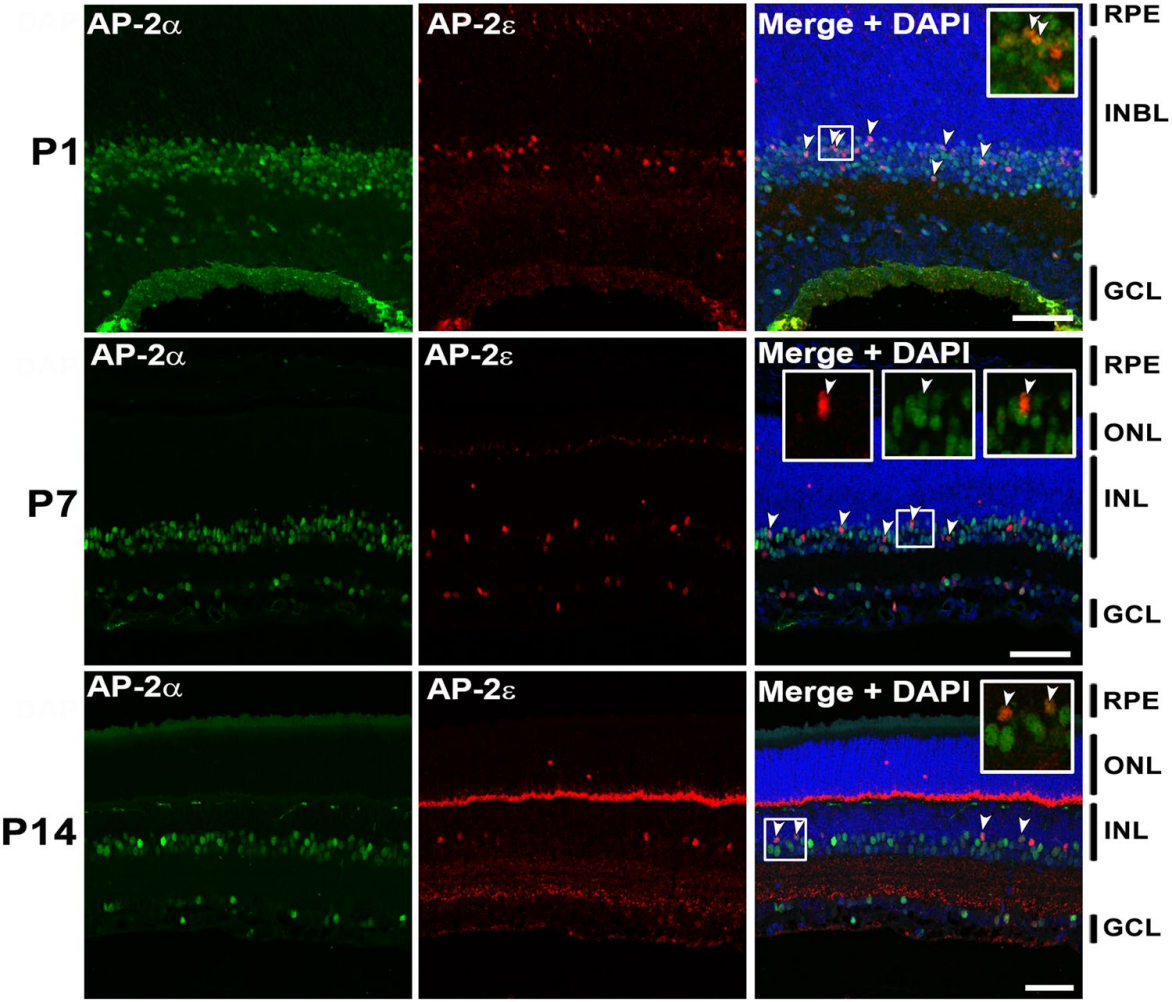
Co-immunostaining of AP-2α and AP-2ε in P1, P7 and P14 mouse retina. Tissues were co-immunostained with rabbit anti-AP-2ε (red) and mouse anti-AP-2α (green). DAPI was used to stain nuclei. Merged images show co-localization of AP-2α and AP-2ε (yellow/orange color). Arrowheads point to cells co-expressing AP-2α and AP-2ε. Insets show a magnified view of designated areas. In P7, the arrowhead in the three insets points to the same cell co-expressing AP-2α and AP-2ε in the red, green and combined red/green channels. Abbreviations: RPE, retinal pigmented epithelium; INL, inner nuclear layer; ONL, outer nuclear layer; INBL, inner neuroblastic layer; GCL, ganglion cell layer. Scale bars = 50 µm.

**Figure 4:**
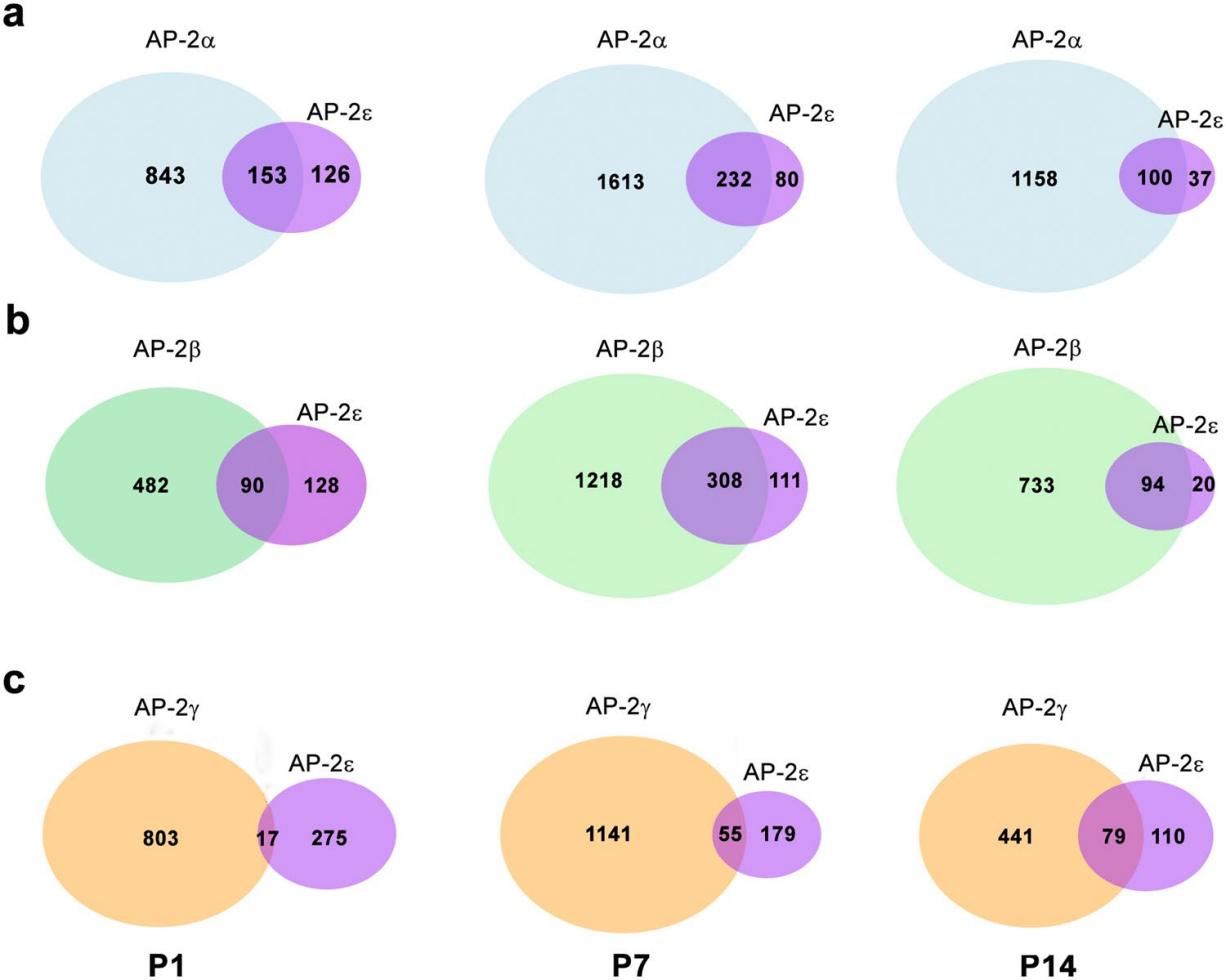
Quantification of AP-2 expression data obtained from co-immunofluorescence analysis. Venn diagrams depict number of cells expressing a particular AP-2 with overlapping areas indicating the number of cells that express both AP-2s. (**a**) Cells expressing AP-2α, AP-2ε, and both AP-2α and AP-2ε. (**b**) Cells expressing AP-2β, AP-2ε, and both AP-2β and AP-2ε. (**c**) Cells expressing AP-2γ, AP-2ε, and both AP-2γ and AP-2ε. Counts were obtained from 2 eyes at P1 and P14 and 3 eyes at P7. The size of each oval is representative of the number of cells. RStudio software was used to plot Venn diagrams.

**Figure 5:**
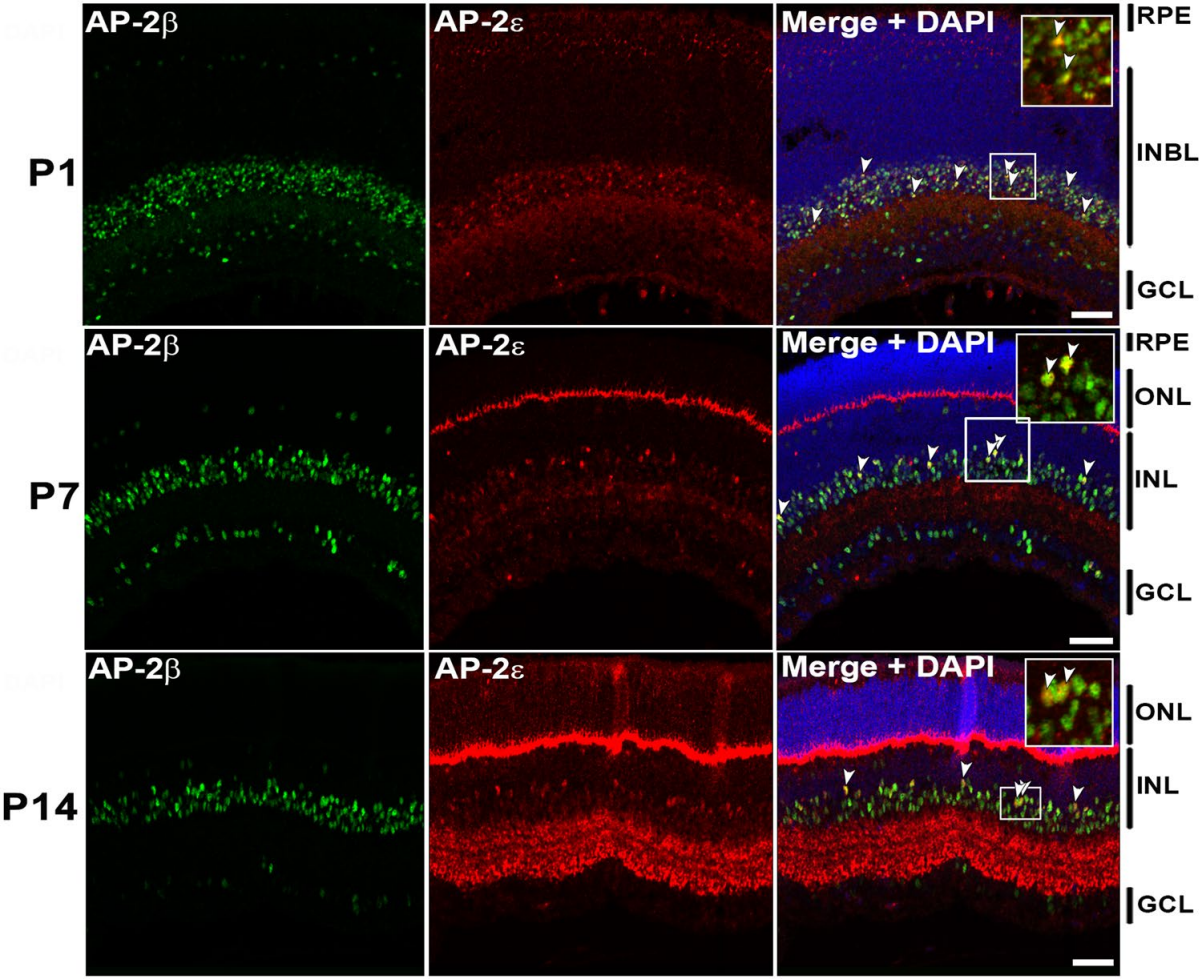
Co-immunostaining of AP-2β and AP-2ε in P1, P7 and P14 mouse retina. Tissues were immunostained sequentially with rabbit anti-AP-2ε (red) and rabbit anti-AP-2β (green). Tyramide signal amplification (TSA) with multiplex capability allowed co-detection of AP-2ε and AP2β (the procedure is explained in Materials and Methods). DAPI was used to stain nuclei. Merged images show co-localization of AP-2β and AP-2ε (yellow). Arrowheads point to cells co-expressing AP-2β and AP-2ε. Insets show a magnified view of designated areas. Abbreviations: RPE, retinal pigmented epithelium; INL, inner nuclear layer; ONL, outer nuclear layer; INBL, inner neuroblastic layer; GCL, ganglion cell layer. Scale bars = 50 µm.

Immunofluorescence analysis was carried out to determine whether AP-2ε is co-expressed with other AP-2 family members at P1 (2 pups), P7 (3 pups) and P14 (2 pups) in mouse retina. Sections from P1, P7 and P14 mouse eyes were first co-immunostained with antibodies to AP-2α and AP-2ε. Considerably fewer AP-2ε-positive cells were observed compared to AP-2α-positive cells (Fig. 3). The majority of AP-2ε-positive cells co-expressed AP-2α (54.8% at P1; 74.4% at P7; 72.9% at P14) (Figs 3, 4a). Similar results were obtained with AP-2β (41.2% at P1; 73.5% at P7; 82.5% at P14) (Figs 5, 4b). Thus, retinal differentiation is accompanied by increased co-expression of AP-2ε with AP-2α and AP-2β.

**Figure 6:**
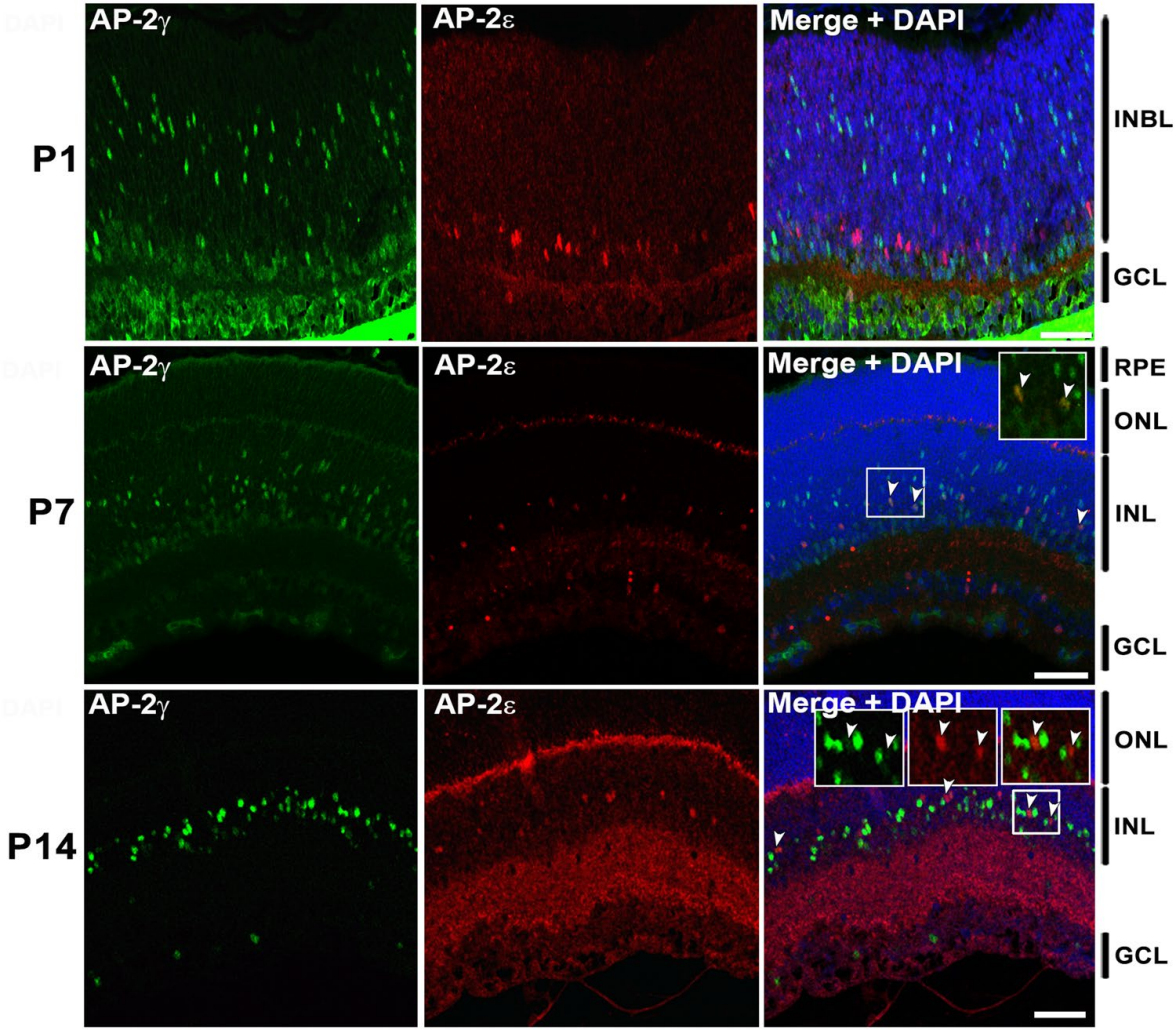
Co-immunostaining of AP-2γ and AP-2ε at P1, P7 and P14 in mouse retina. Tissues were immunostained sequentially with rabbit anti-AP-2ε (red) and mouse anti-AP-2γ (green). DAPI was used as a nuclear stain. Merged images show co-localization of AP-2γ and AP-2ε (yellow/orange). Arrowheads point to cells co-expressing AP-2γ and AP-2ε. Insets show a magnified view of designated areas. In P14, the two arrowheads in the three insets point to the same two cells co-expressing AP-2γ and AP-2ε in the red, green and combined red/green channels. Abbreviations: RPE, retinal pigmented epithelium; INBL, inner neuroblastic layer; ONL, outer nuclear layer; INL, inner nuclear layer; GCL, ganglion cell layer. Scale bars = 50 µm.

AP-2γ and AP-2ε co-immunostaining revealed expression patterns different from that described for AP-2α and AP-2β, with little co-expression observed at early developmental stages. At P1, only 3.9% AP-2ε-positive cells co-expressed AP-2γ. At P7, 23.5% AP-2ε-positive cells co-expressed AP-2γ. At P14, 41.7% AP-2ε-positive cells co-expressed AP-2γ (Figs 6, 4c). The relatively high percentages of AP-2ε-positive cells co-expressing AP-2α (72.9%), AP-2β (82.5%) and AP-2γ (41.7%) at P14 suggest that a significant proportion of AP-2ε-positive amacrine cells co-express three or more AP-2’s.

As we observed a few AP-2ε-positive cells in the ganglion cell layer, we co-immunostained mouse tissue sections with anti-AP-2δ (ganglion cell-specific) and anti-AP-2ε antibodies. There was no co-localization of AP-2δ and anti-AP-2ε at any of the stages analyzed (P1, P7 and P14) (data not shown). These combined data demonstrate complex spatial relationships between AP-2ε and AP-2α, β, γ in the developing retina, with strongest associations observed with AP-2α and AP-2β at P7 and P14. The limited overlap in AP-2ε and AP-2γ co-expression at P1 suggests that there is little need for AP-2γ/AP-2ε-positive amacrine cells at early stages of retinal development.

### AP-2ε is expressed in GABAergic amacrine cells

**Figure 7:**
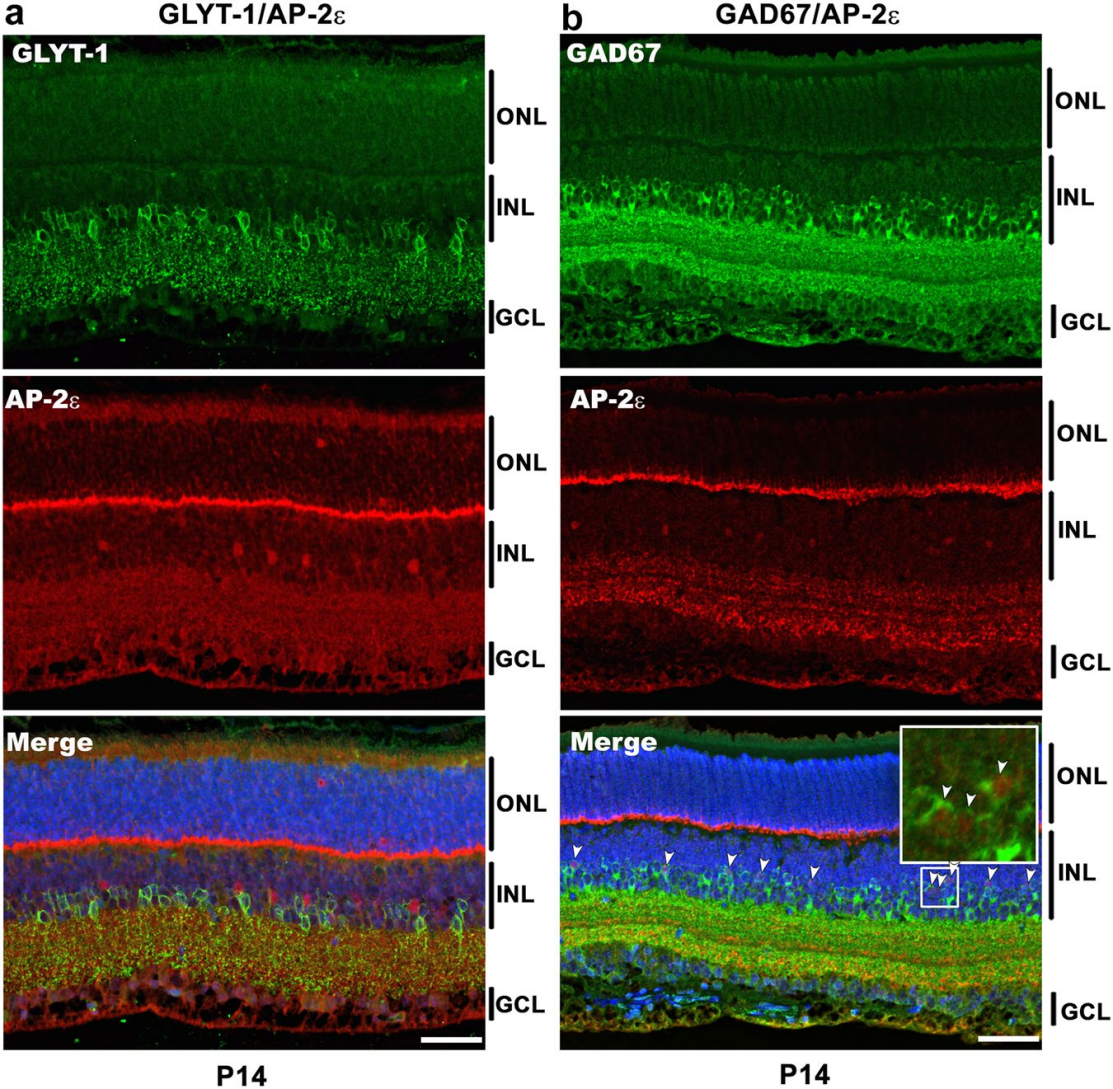
Co-immunofluorescence showing AP-2ε expression in glycinergic and GABAergic amacrine cells. (**a**) P14 mouse retina tissue sections were co-immunostained with anti-AP-2ε antibody (red) and anti-GLYT-1 antibody (glycinergic amacrine cell marker; green). Little, if any, co-immunostaining was observed with the anti-GLYT-1 antibody. (**b**) P14 mouse retina tissue sections were co-immunostained with anti-AP-2ε antibody (red) and anti-GAD67 antibody (GABAergic amacrine cell marker; green). As observed in the merged diagram (bottom panel), most of the AP-2ε positive cells are GAD67 positive. The inset shows a magnified view of the designated area. DAPI was used to stain nuclei. Abbreviations: ONL, outer nuclear layer; INL, inner nuclear layer; GCL, ganglion cell layer. Size bars = 50 µm.

There are two major categories of amacrine cells based on the neurotransmitter used to transmit signals across the retina: glycinergic and GABAergic^40,41^. Previous studies have shown that AP-2α and AP-2β-positive cells can be either glycinergic or GABAergic^26^. We carried out immunofluorescence studies to determine whether AP-2ε is preferentially expressed in glycinergic or GABAergic amacrine cells. Anti-GLYT1 (glycinergic) and anti-GAD67 (a biosynthetic enzyme for GABA) antibodies were used to identify the two different categories of amacrine cells. Examination of P14 mouse retina tissue sections revealed no co-expression of AP-2ε with GLYT1 (Fig. 7a). On the other hand, virtually every AP-2ε-positive cell expressed GAD67, although AP-2ε-positive cells represented a small fraction of GAD67-positive cells (~10%) (Fig. 7b). These results indicate that the amacrine cells that express AP-2ε are GABAergic.

We also examined AP-2ε/GAD67 co-immunostaining in the ganglion cell layer. The observed co-immunostaining of AP-2ε and GAD67 confirmed that the few AP-2ε-positive cells in the ganglion cell layer are indeed displaced amacrine cells (Supplementary Fig. S3).

### Co-expression analysis of AP-2 transcription factors using single cell RNA sequencing data

Macosko *et al*.^42^ carried out single-cell Drop-seq analysis of 44,808 cells isolated from P14 mouse retina and obtained 39 transcriptionally distinct clusters ranging in size from 50 to 29,400 cells. Ganglion cells, cones, rods, horizontal, Müller glia and astrocytic cells were each placed in a single cluster, and bipolar cells in 8 clusters. Amacrine cells were placed in 21 clusters (clusters 3 to 23): 12 GABAergic, 5 glycinergic, 1 excitatory based on glutamate transporter *Slc17a8* expression and 3 with low levels of GABAergic, glycinergic and glutamatergic markers. Subgrouping of the GABAergic and glycinergic clusters was based on differential expression of known amacrine markers. Approximately 10% of sequenced single cells (~4,400 of 44,808 cells) were classified as amacrine cells.

We examined 3,711 cells with complete sequencing data for expression of AP-2 family members. *AP-2ε* RNA sequences were found in 133 cells, 108 of which belonged to two clusters: cluster 5 (21/68 cells) and 9 (87/275 cells) (Table 1). Of the remaining 25 AP-2ε-positive cells, 24 belonged to three clusters: cluster 8 (14/125 cells), cluster 12 (3/224 cells) and cluster 15 (7/57 cells). Next, we examined co-expression of *AP-2ε* RNA with that of the other AP-2s in the same cluster. In cluster 5, 58.8% (40/68), 5.88% (4/68) and 20.59% (14/68) of amacrine cells were positive for AP-2α, AP-2β and AP-2γ, respectively, suggesting the possibility of significant overlap in AP-2α and AP-2ε expression for this particular subset of amacrine cells. For cluster 9, 68.7% (189/275) of cells were positive for AP-2α, 84% (231/275) for AP-2β, and 17.5% (48/275) for AP-2γ indicating at least some overlap with AP-2β, and likely overlap with AP-2α in this cluster (Table 1).

**Table 1:** Twenty-one previously defined clusters of amacrine cells numbered 3 to 23 were analyzed for expression of AP-2 family members.

Cluster	Count	AP-2α	%	AP-2β	%	AP-2γ	%	AP-2ε	%	Co-expression
**3**	233	77	*33.05*	179	*76.82*	0	0	0	0	**AP-2α/β**
**4**	67	6	8.96	1	1.49	0	0	0	0	—
**5**	68	40	*58.82*	4	5.88	14	20.59	21	*30.88*	***AP-2α/ε***
**6**	182	95	*52.2*	58	*31.87*	65	*35.71*	1	0.55	**AP-2α/β/γ**
**7**	257	55	21.4	38	14.79	148	*57.59*	0	0	**AP-2γ**
**8**	125	58	*46.4*	82	*65.6*	14	11.2	14	11.2	**AP-2α/β**
**9**	275	189	*68.73*	231	*84*	48	17.45	87	*31.64*	***AP-2α/β/ε***
**10**	166	78	*46.99*	135	*81.33*	11	6.63	0	0	**AP-2α/β**
**11**	189	42	22.22	136	*71.96*	5	2.65	0	0	**AP-2β**
**12**	224	57	25.45	157	*70.09*	5	2.23	3	1.34	**AP-2β**
**13**	41	31	*75.61*	39	*95.12*	0	0	0	0	**AP-2α/β**
**14**	95	62	*65.26*	92	*96.84*	0	0	0	0	**AP-2α/β**
**15**	57	32	*56.14*	50	*87.72*	2	3.51	7	12.28	**AP-2α/β**
**16**	211	35	16.59	198	*93.84*	2	0.95	0	0	**AP-2β**
**17**	328	0	0	10	3.05	200	*60.98*	0	0	**AP-2γ**
**18**	75	1	1.33	4	5.33	41	*54.67*	0	0	**AP-2γ**
**19**	115	9	7.83	5	4.35	3	2.61	0	0	—
**20**	342	1	0.29	6	1.75	0	0	0	0	—
**21**	224	0	0	8	3.57	1	0.45	0	0	—
**22**	228	57	25	188	*82.46*	0	0	0	0	**AP-2β**
**23**	209	82	*39.23*	192	*91.87*	2	0.96	0	0	**AP-2α/β**

The table shows numbers of cells in each cluster and the number and percentage of cells expressing AP-2α, AP-2β, AP-2γ or AP-2ε. The clusters which have >30% cells for a particular AP-2 are Italic. The last column indicates possible combinations of AP-2s expressed in each cluster based on percentage of cells (30% cut-off) expressing specific AP-2s. Co-expression clusters that include AP-2ε are Italic.

**Figure 8:**
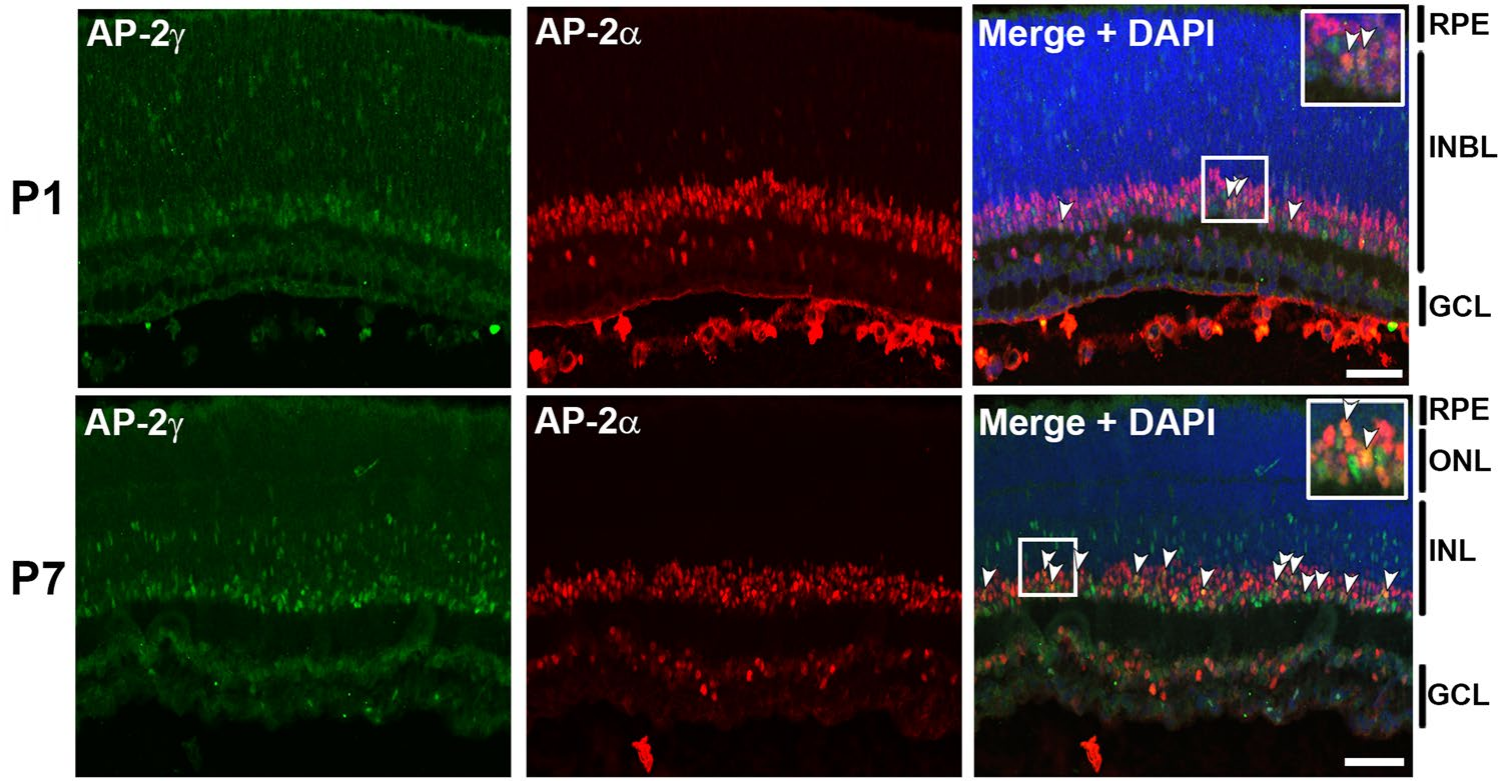
Co-immunostaining of AP-2α and AP-2γ in P1 and P7 mouse retina. Tissues were co-immunostained with mouse anti-AP-2α (red) and mouse anti-AP-2γ (green) antibodies. Tyramide signal amplification (TSA) with multiplex capability allowed co-detection of AP-2α and AP2γ (the procedure is explained in Materials and Methods). DAPI was used to stain the nuclei. Merged images show co-localization of AP-2α and AP-2γ (yellow/orange; indicated by arrowheads). The insets show magnified views of the designated areas. DAPI was used to stain nuclei. Abbreviations: RPE, retinal pigmented epithelium; INL, inner nuclear layer; ONL, outer nuclear layer; INBL, inner neuroblastic layer; GCL, ganglion cell layer. Scale bars = 50 µm.

In keeping with previously reported data on AP-2 expression in mouse retina^26^, there were clusters (e.g. cluster 6) with elevated levels of AP-2α, AP-2β and AP-2γ sequences. As co-expression of AP-2α and AP-2γ has not previously been reported in the literature, we tested this *in silico* finding by co-immunofluorescence analysis using anti-AP-2γ and AP-2α antibodies. Our analysis revealed only a few AP-2α/AP-2γ co-expressing cells in P1 mouse retina, with the number of AP-2α/AP-2γ-co-expressing cells increasing at P7 compared to P1 (Fig. 8). Notably, AP-2γ-positive cells located in the middle of the inner nuclear layer, presumably migrating amacrine cells, were exclusively negative for AP-2α (Fig. 8). The pattern of expression of AP-2γ compared to AP-2α suggests that AP-2γ is expressed in migrating cells committed to the amacrine lineage as well as differentiated amacrine cells, and that AP-2γ-expressing amacrine cells differentiate later than AP-2α-expressing amacrine cells. This interpretation of our results is in agreement with Drop-seq data showing amacrine clusters that were positive for AP-2γ, with no or very little AP-2α or AP-2β (clusters 17 and 18). Thus, the Drop-seq data for the different members of the AP-2 family suggest overlapping expression patterns that are aligned with co-immunofluorescence data obtained by us and others.

### AP-2ε RNA is expressed in retinoblastoma cell lines

**Figure 9:**
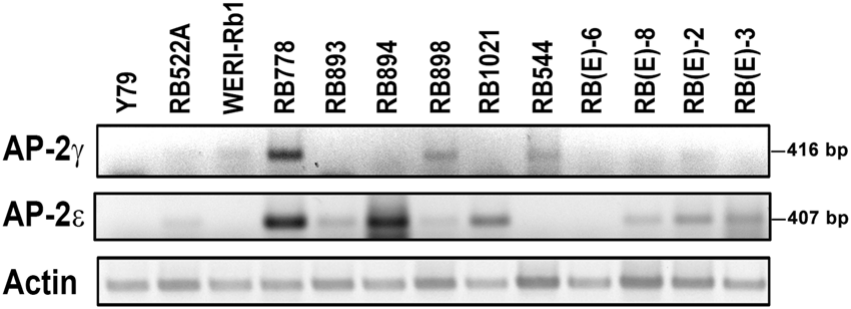
Semi-quantitative RT-PCR showing expression of *AP-2γ* and *AP-2ε* in RB cells. RT-PCR was carried out using cDNAs obtained from 13 RB cell lines. Sizes of RT-PCR products are indicated on the right. Actin was used as the loading control. Full length blots are shown in Supplementary Fig. S1.

We have previously shown that *AP-2α* RNA is expressed in retinoblastoma cell lines^43^. However, retinoblastoma cells do not express *AP-2β* RNA^43^. To further investigate expression of amacrine lineage-specific AP-2s in retinoblastoma cells, we examined the expression of *AP-2γ* and *AP-2ε* RNA in 13 retinoblastoma cell lines. Semi-quantitative RT-PCR analysis revealed expression of *AP-2γ* and *AP-2ε* in subsets of retinoblastoma lines (Fig. 9 and Supplementary Fig. S1). With the exception of RB778 cells which were positive for both *AP-2γ* and *AP-2ε*, there was a trend towards mutual exclusion for these two AP-2s. These results provide further support for a link between RB and amacrine cells and suggest that AP-2 expression patterns in retinoblastoma cell lines mimic developmentally-regulated amacrine cell differentiation patterns.

## Discussion

Four members of the AP-2 family (α, β, γ and δ) have previously been shown to be expressed in subsets of cells in the retina. In this study, we demonstrate that the fifth member of the AP-2 family, AP-2ε, is also expressed in vertebrate retina. Like AP-2α, AP-2β and AP-2γ, AP-2ε is specifically expressed in a subset of amacrine cells. The AP-2ε expression pattern is similar in chicken, mouse and human, although peak expression is observed at an earlier developmental stage in mouse (P1) compared to chicken (E10).

AP-2ε has previously been shown to have a limited expression pattern during mouse embryogenesis compared to AP-2α, AP-2β and AP-2γ. AP-2ε is mainly expressed in developing brain and spinal cord in mouse, with highest levels in the olfactory bulb^11^. AP-2ε is no longer detected in the olfactory bulb by P14^11^. *AP-2ε*-knockout mice have an abnormal olfactory bulb architecture^21^, with defects in lamination of projection neurons and their associated axons. Surprisingly, *AP-2ε−/−* mice can still sense odors, suggesting that other members of the AP-2 family may be compensating for loss of AP-2ε in the olfactory bulb^21^.

The expression pattern of AP-2ε in amacrine cells is more restricted than that of the other AP-2s. For example, both AP-2α and AP-2β are widely distributed in amacrine cells, with AP-2α/AP-2β co-expression observed in a high percentage of cells^27^. AP-2γ is primarily found in a subset of amacrine cells distinct from those expressing AP-2α and AP-2β (AP-2β/AP-2γ co-localization is described in Bassett *et al*.^26^; AP-2α/AP-2γ co-localization is shown in Fig. 8). Although ocular abnormalities have not yet been reported for *AP-2ε−/−* mice, this may be due to the relatively small number of cells expressing AP-2ε in the retina. As a case in point, it was only upon detailed analysis of the retina and visual centers of the brain that an ocular phenotype was identified in *AP-2δ* knockout mice^20^. Furthermore, as described for AP-2α and AP-2β^26^, other members of the AP-2 family may compensate for loss of AP-2ε expression.

Structurally, AP-2 proteins have a helix-span-helix dimerization domain at the carboxy terminal region preceded by a basic region. The helix-span-helix motif along with the central basic region constitute the DNA binding domain^44,45^. The DNA binding domain of AP-2ε is evolutionary conserved and is highly similar to that of the other AP-2s suggesting that these transcription factors bind to similar AP-2 recognition elements. In agreement with this, AP-2ε binds to the consensus AP-2 recognition element GCCNNNGGC as either a homodimer or heterodimer with other members of the AP-2 family^46,47^. The N-terminus contains the activation domain which is generally less conserved between different AP-2 proteins except for a proline-rich region found in all AP-2s except AP-2δ^44–47^. These similarities and differences in the structure of AP-2 proteins may affect protein-protein and protein-DNA interactions, thereby determining AP-2 target gene specificity. For example, AP-2α, AP-2β and AP-2γ can all bind as either homodimers or heterodimers to an AP-2 recognition site in the c-erbB2 promoter; however, AP-2α and AP-2γ are four times more active than AP-2β at activating a c-erbB2-driven reporter construct^48^. Considering that: (i) there are four members of the AP-2 family expressed in amacrine cells with distinct and overlapping patterns, (ii) AP-2 can function as either homodimers or heterodimers, and (iii) there are at least 33 amacrine subtypes in mammalian retina^32,33^, AP-2s could play key roles in the determination of amacrine subtype-specific functions in the retina.

Only a few genes have been identified as AP-2ε target genes in mammals, including *ITGA10* encoding integrin α10 (important for chondrocyte differentiation)^49^, *COL2A1* (involved in modulation of cartilage development)^50^, *DKK4* (associated with resistance to chemotherapy in colon cancer)^51^, and *CDKNA1* encoding p21WAF1 (identified in neuroblastoma cells)^52^. The *Mmp13* gene has also been shown to be upregulated in adult *AP-2ε−/−* mice, although it’s not known whether *Mmp13* is a direct target of AP-2ε^53^. Similarly, *Xenopus* AP-2ε activates neural crest-specific genes *Snail2* and *SOX10*^54^, and zebrafish AP-2ε activates *kita* expression in melanophores and helps promote melanophore differentiation^55^. Additional putative AP-2ε target genes come from cDNA microarray analysis of colorectal cancer cells that either express or don’t express AP-2ε^51^. Of the top 50 genes identified in this cDNA microarray, one was previously shown to be expressed in amacrine cells in chick retina: Tenascin C (*TNC*)^56^. Cluster analysis of Drop-seq data, dividing amacrine cells into 3 clusters [group 1 including clusters 5 and 9 (>30% AP-2ε-positive cells), group 2 including clusters 8 and 15 (10–15% AP-2ε-positive cells), group 3 including the remaining clusters with few or no AP-2ε-positive cells], revealed no correlation with any of the putative AP-2ε target genes mentioned above. A next step would be to compare gene expression in wild-type versus *AP-2ε−/−* retina in an attempt to identify AP-2ε target genes.

Amacrine cells are broadly defined on the basis of the neurotransmitter that they use to transmit signal. There are two main categories of neurotransmitters in amacrine cells: GABAergic (GAT1 positive) and glycinergic (GLYT1 positive)^40,41^. GABAergic amacrine cells are further divided based on whether they express cholinergic (*VAChT* positive) or tachykinin (*Tac1* positive) neurotransmitters. AP-2α, AP-2β and AP-2γ are found in both GABAergic and glycinergic amacrine cells. In contrast, AP-2ε is exclusively found in GABAergic amacrine cells, a result that is in agreement with the Drop-seq data^42^. Thus, AP-2ε may play a specialized role in the regulation of genes involved in fate determination of GABAergic amacrine cells.

A previous study showed that *AP-2α* RNA is expressed in retinoblastoma cells^43^. Transfection of either AP-2α or AP-2β expression constructs in retinoblastoma cells induced apoptosis, suggesting incompatibility with expression of AP-2 amacrine cell differentiation markers and survival in retinoblastoma cells^43^. Our results demonstrating *AP-2γ* and *AP-2ε* expression in retinoblastoma cells further support an amacrine cell lineage for retinoblastoma tumors. The trend towards mutual exclusion of *AP-2ε* and *AP-2γ* RNAs in different retinoblastoma cell lines also supports the idea that retinoblastoma tumors are derived from different amacrine subtypes, in keeping with the low level of overlap between AP-2ε- and AP-2γ-expressing amacrine cells at early stage of retinal differentiation in mouse.

In conclusion, we show that AP-2ε is expressed in a subset of amacrine cells in developing vertebrate retina with peak expression at P1 in mouse and E10 in chick. AP-2ε expression overlaps with the other three AP-2s previously shown to be expressed in amacrine cells, with extensive overlap with AP-2α and AP-2β at all stages tested. Our immunostaining data are in agreement with previously reported sequencing data obtained from single amacrine retinal cells by Drop-seq. Expression of four AP-2s in amacrine cells suggests complex and cell-specific roles for this family of transcription factors in determining the identity and/or function of amacrine cell subtypes.

## Materials and Methods

### Ethics statements

Ethics approval for the collection of human fetal retina was obtained from the Health Research Ethics Board of Alberta (HREBA) – protocol 17561 (new IRISS study number HREBA.CC-17-0475). For human fetal retina (collected more than 20 years ago), the need for consent was waived as the tissue was collected under a general protocol that did not require any information regarding the patient. Ethics approval for collection of mouse retina after euthanasia was obtained from the Cross Cancer Institute Animal Care Committee – protocol AC 16226. All methods used for collection of retinal tissue were carried out in accordance with human ethics and Canadian Council on Animal Care (CCAC) Guidelines and Policies, with all experimental procedures approved by HREBA and by CCI Animal Care Committee under protocols 17561 and AC 16226, respectively. Chick retinal tissue was collected from euthanized animals as part of previous studies^28,57^. None of the studies reported in the manuscript involved live vertebrates.

### Semi-quantitative and quantitative RT-PCR

RNA was purified from at least two different batches of chick retina [embryonic day E5, E7, E10 and E15] and mouse retina [E16.5, post-natal (P)1, P14 and adult]. RNA was reverse transcribed using oligo(dT) and Superscript reverse transcriptase II (Invitrogen). The following primers were used for semi-quantitative RT-PCR analysis of mouse and chicken retina: mouse AP-2ε (forward primer: 5′-GTTGCTCAGCTCAACATCCA-3′; reverse primer: 5′-CTGAGCCATCAAGTCTGCAA-3′), and chicken AP-2ε (forward primer: 5′-GCTCCACACCAGGAAGAACATG-3′; reverse primer: 5′-CAT CAA ACT GGC TCA TTT TC-3).

Y79 and WERI-Rb1 retinoblastoma cell lines were obtained from the American Type Culture Collection. RB522A, RB778, RB893, RB894, RB898, RB1021, RB544 cell lines were established by Dr. Brenda Gallie, Department of Medical Genetics, University of Toronto, Canada. RB(E)-2, RB(E)-3, RB(E)-6 and RB(E)-8 cell lines, as well as RNA preparation, have been previously described^43^. The following primers were used for RT-PCR analysis of retinoblastoma cells: human AP-2ε (forward primer: 5′-CAATGTGACGCTGCTGACTT-3′; reverse primer: CACTGCCCACACTGCTTAG-3′), and human AP-2γ (forward primer: 5′-AAAGCCGCTCATGTGACTCT-3′; reverse primer: TGGTCTCCAGGGTTCATGT-3′). Quantitative RT-PCR was carried out using the SYBR green based qPCR system (Applied Biological Materials Inc. Canada) and analyzed on an ABI 7900HT PCR system, with primers designed to amplify a 150 bp region of *AP-2ε* (forward primer: 5′-ATTGCAGGCGATAGATGACC-3′; reverse primer: 5′-GAGCAGAAGACCTCACTGG-3′).

### *In situ* hybridization

Tissue sections (7–8 μm) were prepared from E16.5, P1, P7, P15.5 mouse retina, as well as E10 chick retina. Tissue sections were fixed in 4% paraformaldehyde, and incubated with DIG-labelled probes overnight at 55 °C, as previously described^58^. Tissue sections were then washed and digested with ribonuclease A. The signal was detected with anti-DIG antibody using NBT and BCIP as substrates^24^. For *in situ* hybridization probes, we PCR amplified a 700 bp mouse *AP-2ε* cDNA fragment and a 560 bp chicken *AP-2ε* cDNA fragment. cDNAs were cloned into the pBluescript vector and pGEM-T Easy vector, respectively. The constructs were linearized and electrophoresed in a polyacrylamide gel. Bands were cut out, electrophoresed out of the gel and the DNA extracted with phenol and chloroform. Probes were generated using T3 and T7 RNA polymerases and digoxygenin (DIG)-labeling mix, as specified by the manufacturer (Roche). The probes were quantified by comparing labeling intensity to a control probe supplied by manufacturer. Image acquisitions were made using a Zeiss Axioskop2 Plus microscope and AxioVision 4.7.1 software.

### Immunostaining

Immunohistochemistry and immunofluorescence analyses were carried out as previously described^43,59^. Mouse P1 (from 2 pups), P7 (from 3 pups) and P14 (from 2 pups) retinas and human fetal retina tissue at 17 weeks gestation were fixed in formalin and paraffin-embedded. Note that mouse retina tissue at P14–16 is roughly equivalent, with most cells in the retina being fully differentiated by P11^36^. Tissue sections were deparaffinized in xylene, rehydrated and microwaved in a pressure cooker for 20 min for antigen retrieval. The rabbit anti-AP-2ε antibody (1:1,500, generated by Dr. Markus Moser, Max Plank Institute of Biochemistry) was used for immunohistochemistry. The following antibodies were used for immunofluorescence analysis: anti-AP-2α, mouse monoclonal antibody (1:400, 3B5, Developmental Studies Hybridoma Bank developed under the auspices of the NICHD and maintained by the University of Iowa)^26^, anti-AP-2β, rabbit polyclonal antibody (1:1,000, #2509, Cell Signaling Technology)^26^, anti-AP-2γ, mouse monoclonal antibody (1:200, 6E4/4, Santa Cruz Biotechnology)^26^, and anti-AP-2ε, rabbit polyclonal antibody (1:1,500, described above). GABAergic amacrine cells were immunostained with anti-GAD67, mouse monoclonal antibody (1:3000, MAB5406, Chemicon International). Glycinergic amacrine cells were immunostained with anti-GLYT1, goat polyclonal antibody (1:6000, AB1770, Millipore).

For co-immunofluorescence of same species anti-AP-2β and anti-AP-2ε rabbit antibodies, we used the Tyramide Signal Amplification (TSA) kit (PerkinElmer). Tissue sections were first immunostained with anti-AP-2ε antibody followed by goat anti-rabbit HRP-conjugated secondary antibody (DAKO). Cy3-conjugated tyramide was then applied, resulting in a tyramide-protein-antibody complex, with HRP activating bound tyramide. The protein-antibody complex was then heat-denatured in sodium citrate, leaving the Cy3-conjugated tyramide intact. Next, tissue sections were immunostained with AP-2β primary antibody followed by Alexa 488-conjugated secondary antibody. Immunofluorescence images were captured on a Zeiss LSM710 confocal laser scanning microscope with a plan-Apochromat 20X lens using ZEN software.

To ensure AP-2 antibody specificity, HeLa cells were transfected with each of the five AP-2 expression constructs in p3xFLAG-CMV vector. Cells were harvested, and total cell lysates were prepared using RIPA buffer. Lysates were electrophoresed through a 10% SDS-PAGE gel and western blot analysis carried out using antibodies to each of the five AP-2s.

### Analysis of AP-2 expression in mouse retinal cells

We utilized the merged expression data set generated by Drop-seq of single P14 mouse retinal cells (GEO accession viewer GSE63472)^42^. Individual cells were grouped as per the 39 clusters defined by Macosko *et al*.^42^, and further analysis was performed on those clusters identified as amacrine cell types (clusters 3 to 23, comprising a total of 3,711 individual cells). These cells were then scored as positive or negative for expression of AP-2α, AP-2β, AP-2γ or AP-2ε. A more detailed analysis of AP-2ε was performed with cell clusters containing >30% AP-2ε-positive cells (high expression or HE, clusters 5 and 9: 343 cells), >10% AP-2ε- positive cells (mid-level expression or ME, clusters 8 and 15: 182 cells), with the remaining cell clusters labelled as low or no expression (NE, 3,186 cells).

### Data availability statement

The datasets analyzed in the current study are from a published study by Macosko *et al*.^42^. These datasets are publicly available through GEO accession viewer repository GSE63472.

## Electronic supplementary material

Supplementary information
