# Supplementary material for: AP-2ε Expression in Developing Retina: Contributing to the Molecular Diversity of Amacrine Cells
Source: Sci Rep. 2018 Feb 21;8:3386. doi: 10.1038/s41598-018-21822-y (PMC5821864; doi:10.1038/s41598-018-21822-y)
Supplement: Supplementary file 1 — Supplementary information [file 41598_2018_21822_MOESM1_ESM.pdf]

AP-2ε Expression in Developing Retina: Contributing to the Molecular Diversity of Amacrine Cells

Saket Jain, Darryl D. Glubrecht, Devon R. Germain, Markus Moser and Roseline Godbout*

Supplementary Information

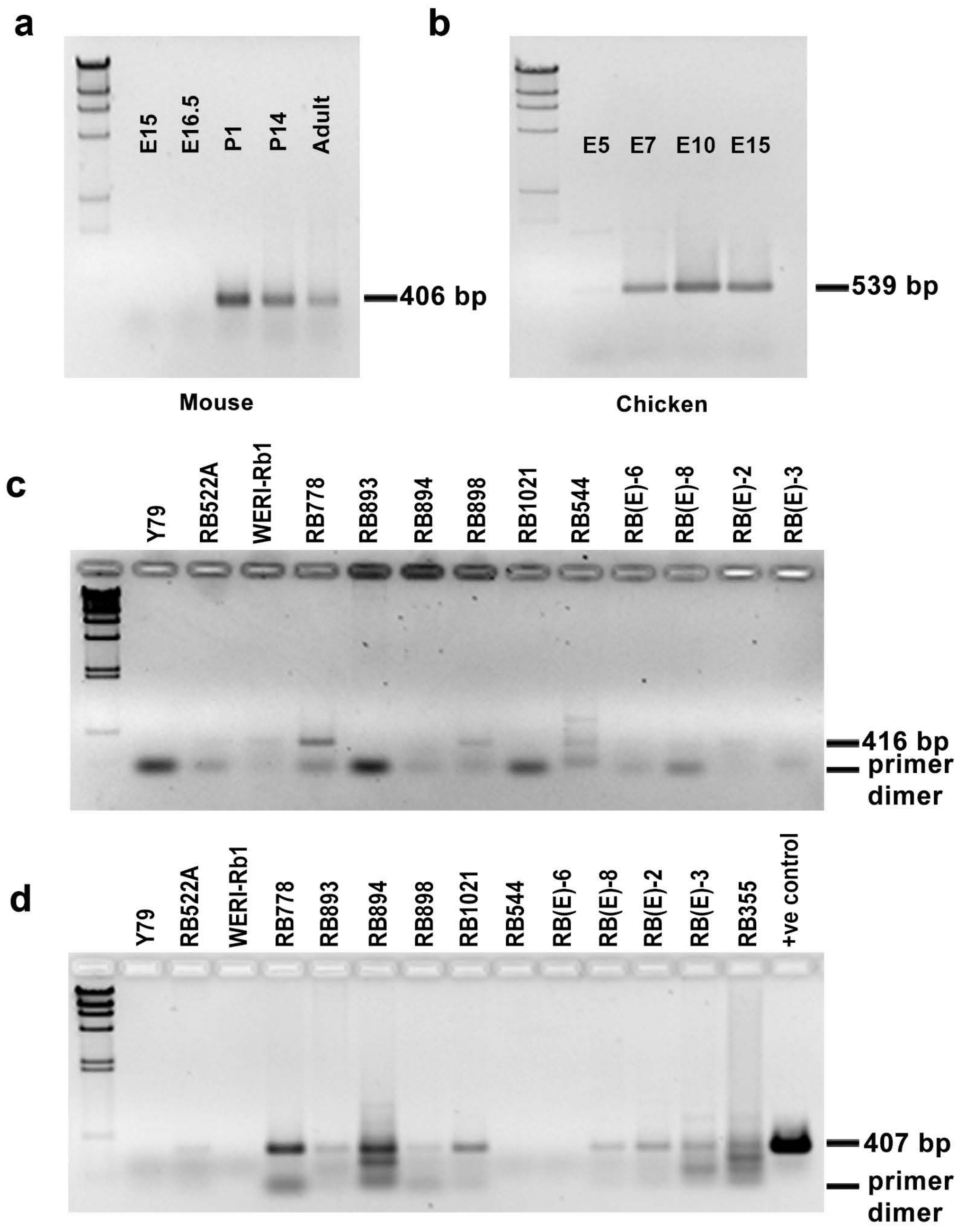

Figure S1: RT-PCR analysis of AP-2ε in retina and retinoblastoma. (a) RT-PCR analysis of AP-2ε in mouse retina at E15, E16.5, P1, P14 and adult. (b) RT-PCR analysis of AP-2ε in chick retina at E5, E7, E10 and E15. (c) RT-PCR analysis of AP-2γ in retinoblastoma cell lines (13 retinoblastoma cell lines in the original figure). (d) RT-PCR analysis of AP-2ε in retinoblastoma cells (14 retinoblastoma cell lines in the original figure). A positive control (U251 malignant glioma cells overexpressing AP-2ε) is included with the analysis of AP-2ε. The sizes (in bp) of the RT-PCR products are shown on the right. (a) and (b) are full-length blots of the data presented in Figs. 1c. (c) and (d) are full-length blots of the data presented in Fig. 9.

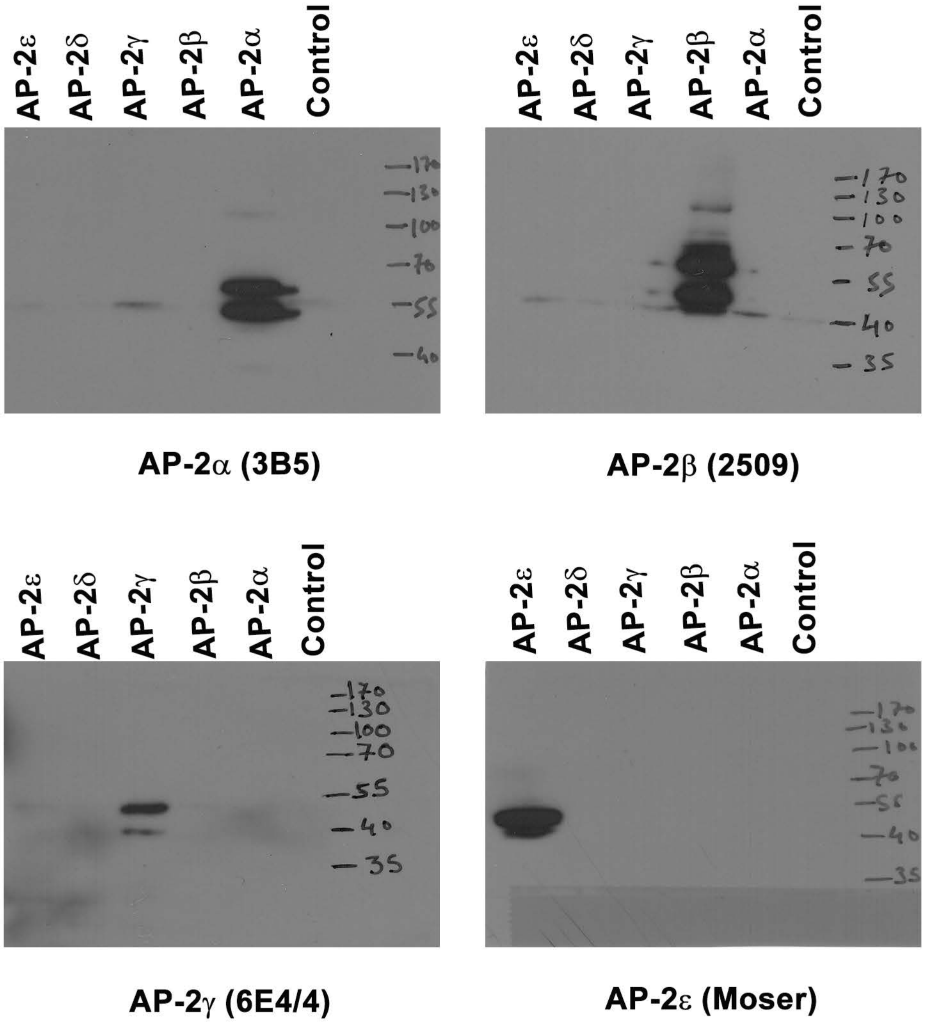

Figure S2: Analysis of specificity of AP-2 antibodies. Western blot analysis of HeLa cells transfected with AP-2α, AP-2β, AP-2γ, AP-2δ or AP-2ε**.** Blots were immunostained with antibodies to AP-2α (a), AP-2β (b), AP-2γ (c) or AP-2ε (d). All the blots shown are original full-length blots. Hand written size markers indicate molecular mass (in kDa).

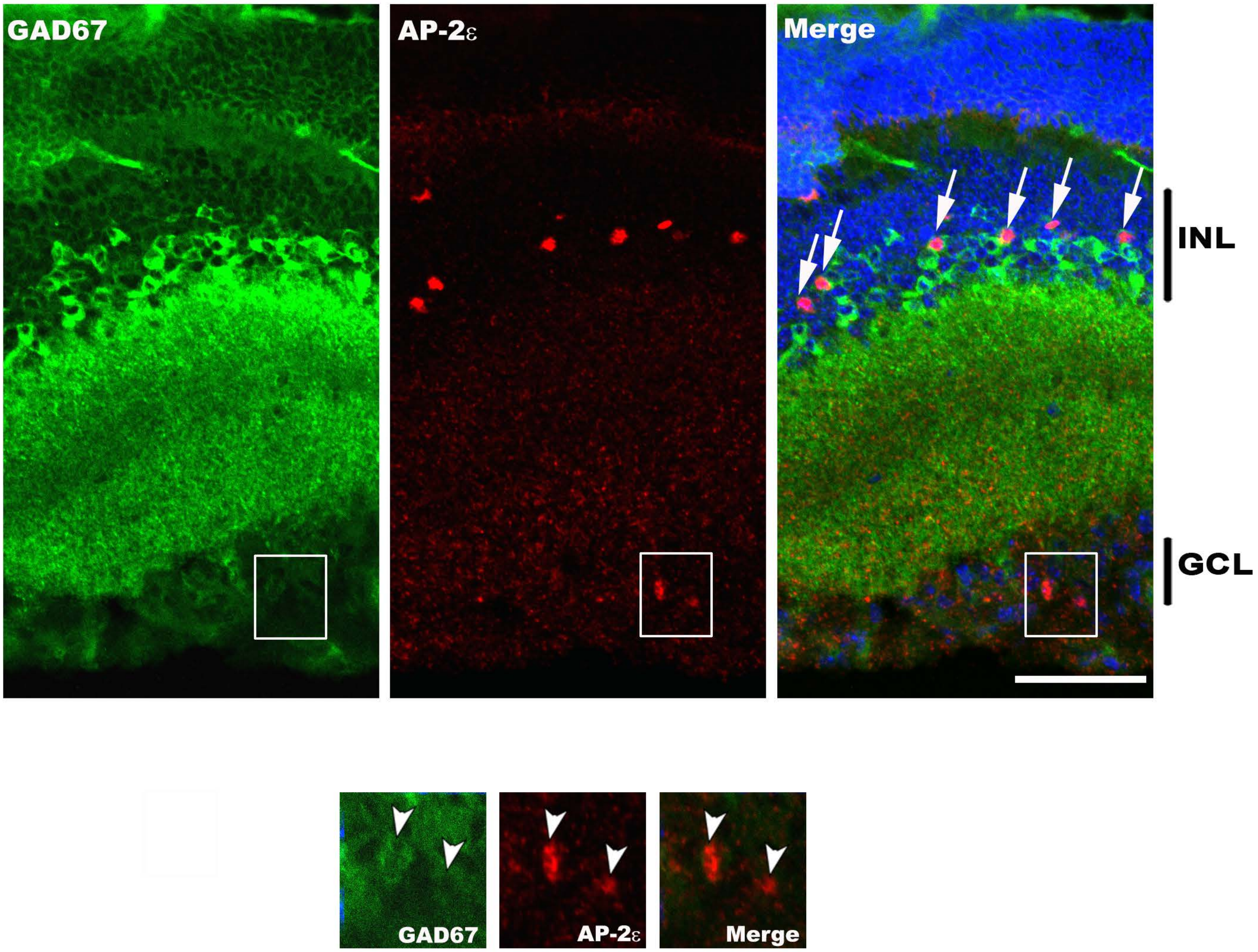

Figure S3: Co-immunostaining of AP-2ε and GAD67 in displaced amacrine cells. P14 mouse retina tissue sections were co-immunostained with anti-AP-2ε (red) and anti-GAD67 antibodies (GABAergic amacrine cell marker; green). A subset of amacrine cells in the inner nuclear layer co-express AP-2ε and GAD67 (indicated by arrows in merged diagram). The rectangles (magnified in the bottom panels) point to two AP-2ε–positive cells in the ganglion cell layer. Both these cells (indicated by arrowheads) co-express GAD67, indicating that they are displaced amacrine cells. DAPI was used to stain nuclei. Abbreviations: INL, inner nuclear layer; GCL, ganglion cell layer. Size bars = 50 μm.
